# Detection and Molecular Characterization of Bovine Leukemia Virus in Egyptian Dairy Cattle

**DOI:** 10.3389/fvets.2020.00608

**Published:** 2020-09-10

**Authors:** Rania Hamada, Samy Metwally, Meripet Polat, Liushiqi Borjigin, Alsagher O. Ali, A. A. A. Abdel-Hady, Adel E. A. Mohamed, Satoshi Wada, Yoko Aida

**Affiliations:** ^1^Photonics Control Technology Team, RIKEN Center for Advanced Photonics, Saitama, Japan; ^2^Department of Animal Medicine, Faculty of Veterinary Medicine, South Valley University, Qena, Egypt; ^3^Laboratory of Global Animal Resource Science, Graduate School of Agricultural and Life Sciences, The University of Tokyo, Tokyo, Japan; ^4^Department of Animal Medicine, Faculty of Veterinary Medicine, Damanhour University, Damanhour, Egypt; ^5^Nakamura Laboratory, Baton Zone Program, RIKEN Cluster for Science, Technology and Innovation Hub, Saitama, Japan; ^6^Department of Surgery, Anaesthesiology and Radiology, Faculty of Veterinary Medicine, South Valley University, Qena, Egypt

**Keywords:** bovine leukemia virus, Egypt, dairy cattle, prevalence, BLV genotype

## Abstract

Bovine leukemia virus (BLV) causes enzootic bovine leukosis (EBL), the most common neoplastic disease in cattle worldwide. The first EBL outbreak in Egypt was reported in 1997. To date, there are few studies regarding BLV diagnosis using only serological detection and no studies investigating the distribution of BLV provirus, which is the retroviral genome integrated into the host genome, in Egypt. The genetic characteristics of Egyptian BLV strains are also unknown. Therefore, we aimed to detect BLV provirus and determine BLV genetic variability among dairy cattle in Egypt. We collected 270 blood samples of dairy cattle from 24 farms located in five provinces in Egypt. Out of the 270 samples, 58 (21.5%) were positive for BLV provirus. Phylogenetic analysis based on 18 420-bp selected sequences out of 50 isolates of the BLV *env*-gp51 gene demonstrated that Egyptian BLV isolates were clustered into genotype-1 and-4, among 11 genotypes detected worldwide. Furthermore, phylogenetic analysis and alignment of the 501-bp sequence of the *env-*gp51 gene revealed that at least six genetically different strains are present in Egypt. Genotype-1 isolates comprised four different strains (G1-a, G1-b, G1-c, and G1-d) and genotype-4 isolates included two different strains (G4-x and G4-y). Moreover, in one farm with 100% infection rate, we identified three isolates of G1-a strain, 35 isolates of G4-x strain, and two isolates of G4-y strain. Overall, this study provides the new report on molecular prevalence of BLV in Egypt and records the coexistence of BLV genotype-1 and-4 in Egyptian cattle.

## Introduction

Bovine leukemia virus (BLV), an oncogenic member of the family *Retroviridae*, genus *Deltaretrovirus*, is closely related to the human T-cell leukemia virus types 1 and 2 (1, 2). It causes enzootic bovine leukosis (EBL), the most common neoplastic disease of cattle globally, and imposes a severe economic loss to the dairy industry (1–3). Most BLV-infected cattle are apparently healthy in the aleukemic stage and approximately one-third can enter the persistent lymphocytosis stage characterized by non-malignant polyclonal expansion of B-lymphocytes; however, only 1–5% develop B-cell leukemia, manifesting clinical signs of lymphoma after a long latency (1, 2). BLV infection can be transmitted both vertical route and horizontal route in addition to the other iatrogenic procedures involving the transfer of infected blood between animals (i.e., dehorning, ear tattooing, rectal palpation, and needle reuse), and is responsible for disease propagation in a herd (4). BLV has spread worldwide via the continual trade of breeding cattle between several countries (3). However, vaccines and effective treatments are not yet available for practical application (1).

BLV proviral load (PVL), which represents the retroviral genome integrated into the host genome (5, 6), correlates strongly with not only BLV infection capacity as assessed by syncytium formation (7, 8), but also EBL progression (7, 9, 10). Additionally, it is a useful index for estimating transmission risk (11, 12). For example, previous reports have posited that as determined by the BLV-CoCoMo-quantitative polymerase chain reaction (qPCR)-2 method, the quantitative measurement of PVL (7, 13), BLV provirus was detected in the milk, nasal mucus, and saliva samples from dairy cattle with PVL >10,000, 14,000, and 18,000 copies/10^5^ cells in blood samples, respectively (11, 12). It has been suggested that these infected cattle may increase the risk of BLV transmission via direct contact with healthy cattle. In contrast, cattle with low PVL are known to prevent natural BLV infection (14).

BLV genome consists of four structural and enzymatic regions that encode genes namely, *gag, pro, pol*, and *env*, along with the pX region that encodes two regulatory, *tax* and *rex*, and two accessory genes, *R3* and *G4*, and is flanked by two identical long terminal repeats (LTRs) at the 5′ and 3′ termini (1, 2). The BLV *env* gene is transcribed as an envelope glycoprotein (Env) protein complex that comprises the surface (gp51) and transmembrane (gp30) proteins (1), which play a major role in the virus lifecycle and entry into the host cell (15). gp51 is the target for neutralizing antibodies (16). The conformational epitopes (F, G, and H) located in the N-terminal half of gp51 are strongly involved in syncytium formation and viral infectivity (15, 16). The C-terminal half contains linear epitopes (A, B, D, and E) mapped with antipeptide antibodies (17). In contrast to gp51, gp30 is poorly immunogenic (1) and therefore, BLV *env-*gp51 sequencing is mainly used for BLV phylogenetic studies (3). At least 11 BLV genotypes have been identified worldwide based on the phylogenetic analysis of *env*-gp51 sequences of BLV after identifying BLV genotype-11 in China (3, 18). Three genotypes of BLV, namely genotype-1,-4, and-6, has been mainly detected worldwide (3).

Egypt is a transcontinental country spanning the northeast corner of Africa and southwest corner of Asia. The bovine sector in Egypt is well-integrated with cropland since there are limited natural pastures. Female cattle and buffaloes are used for milk production, while male animals and infertile females are fattened for meat (19). Native cattle in Egypt are called “Baladi,” literally meaning “local” without genetic subdivisions. They are reared throughout the country, acclimatized to Egyptian conditions, and have a high tolerance to endemic diseases. Due to the low milk productivity of these animals, genetic improvement schemes have involved crossbreeding with exotic high producing cattle breeds such as Holstein, Brown Swiss, and Simmental (20–22), resulting in a large scattered population raised in small- or medium-sized herds by local farmers under the breed name “Mixed,” as described and characterized by the Ministry of Agriculture and Land Reclamation. Egyptian livestock import has increased over the last 10 years, improving the dairy industry performance. Germany, Netherlands, and the United States of America remain the top suppliers of live dairy cattle to Egypt (23).

Sero-epidemiological surveys have shown that BLV infection is widespread in all continents except Europe, where the disease is present only in the eastern European countries (3). However, reports indicate that BLV infection prevalence in the Middle Eastern countries is somewhat lower than that in the other regions of the world (3). In Egypt, the first EBL outbreak occurred in 1997 in Assiut province, Upper Egypt with typical clinical signs of leukosis in a closed herd of Holstein-Friesian cattle imported from Minnesota, USA 8 years earlier (24). Thus, the authors assumed that these cattle were imported as clinically healthy BLV-infected cattle during the herd construction period and the disease progressed to lymphoma after the latent period passed (24). It was believed that this outbreak introduced BLV in Egypt (25). After completely eradicating the infected animals in the herd (24), Egypt was reported to be BLV-free (3, 26). In 2012, a seroprevalence study for BLV infection among dairy cattle in Egypt was conducted, which reported that 15.83% of the tested dairy cattle were BLV-infected (27). Recently, 20.8% BLV seropositivity was reported during the survey for BLV infection among Egyptian cattle (28). These findings indicate that BLV still infects Egyptian cattle. However, to date there has been no reports on BLV genotyping in Egypt.

In this study, we investigated BLV infection prevalence among dairy cattle in five different regions of Egypt to cover representative area of the country to include the northern (Beheira and Damietta provinces), the central (Fayoum province), and the southern (Qena and Luxor provinces) parts using CoCoMo-qPCR-2 assay. Furthermore, we demonstrated that the BLV PVL level varied among different regions of Egypt. Moreover, by conducting the phylogenetic analysis of BLV *env-*gp51 gene sequence, we investigated the six genetically distinct strains present in Egyptian cattle. Overall, this study provides a new evidence for the molecular detection of BLV in Egypt and records the coexistence of BLV genotype-1 and-4 among Egyptian cattle.

## Materials and Methods

### Sample Collection and DNA Extraction

**Figure 1:**
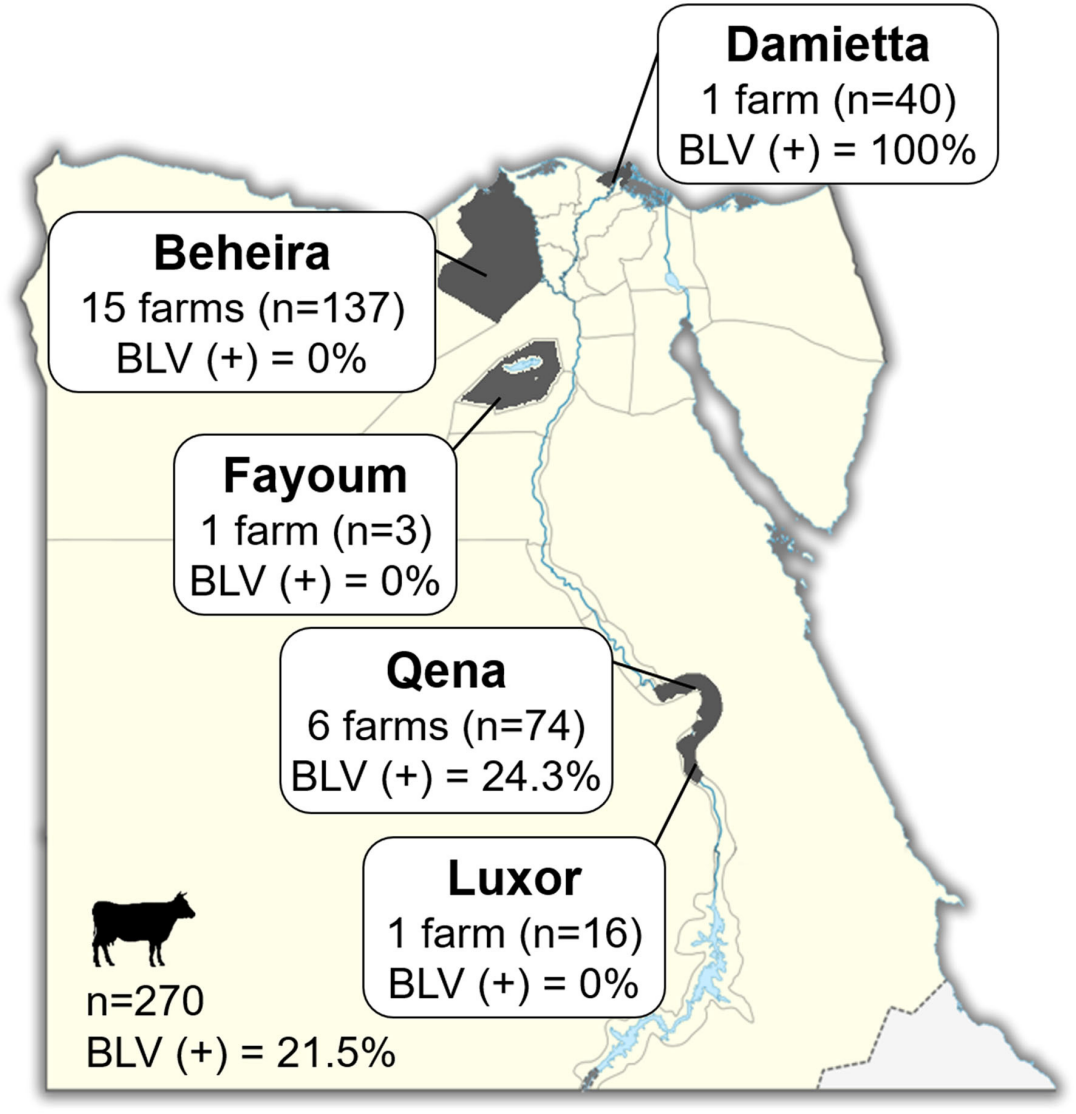
Map of Egypt showing the location of dairy farms for sample collection. Dark gray colored area indicates the geographic location of each region in our study. The number (n; head) of cattle and number (n) of farms in each province and throughout Egypt are indicated in Square. BLV(+) % indicates the BLV prevalence rate.

Blood samples were collected from 270 cattle of 24 different dairy farms located in five provinces in Egypt, namely Luxor, Qena, Fayoum, Beheira, and Damietta, between November 2018 and January 2019 (Figure 1 and Table 1). These cattle comprised various breeds of dairy cattle, such as the Egyptian Native breed, some foreign breeds (Holstein and Simmental), and the Mixed breed, the crossbreed between foreign breeds and Native cattle, and included cattle of both sexes aged 3-months to 12-years (Table 1).

**Table 1:** Detection of BLV infection in five provinces in Egypt as determined by CoCoMo-quantitative PCR (qPCR).

**Province**	**Town**	**Farm ID^**a**^**	**Breed**	**(Positive no. /Tested samples no.) Positive %**
Luxor	EL-Qurna	LQ1	Mixed Native	(0/14) 0.0 (0/ 2) 0.0
Qena	EL-Brahma	QB1 QB2 QB3 QB4 QB5	Mixed Mixed Mixed Native Mixed Native Mixed	(3/10) 30.0 (1/ 4) 25.0 (3/ 5) 60.0 (2/ 5) 40.0 (0/ 2) 0.0 (2/ 4) 50.0 (2/ 5) 40.0
	Qena-city	QC6	Holstein	(5/39) 12.8
Fayoum	Tamia	FT1	Holstein	(0/ 3) 0.0
Beheira	Abu-Elmatamir	BA1 BA2 BA3 BA4 BA5 BA6 BA7 BA8 BA9 BA10 BA11 BA12 BA13 BA14 BA15	Mixed Mixed Mixed Mixed Mixed Mixed Native Mixed Mixed Mixed Mixed Mixed Mixed Mixed Mixed Holstein Simmental	(0/ 9) 0.0 (0/ 2) 0.0 (0/ 7) 0.0 (0/ 2) 0.0 (0/15) 0.0 (0/ 4) 0.0 (0/ 1) 0.0 (0/ 6) 0.0 (0/ 3) 0.0 (0/ 2) 0.0 (0/19) 0.0 (0/ 6) 0.0 (0/ 2) 0.0 (0/ 7) 0.0 (0/ 5) 0.0 (0/43) 0.0 (0/ 4) 0.0
Damietta	El-Zarqa	DZ1	Holstein	(40/40) 100.0
Total farms		24	Total	(58/270) 21.5

a *List of farm ID tested for BLV infection: 17 farms (QB1–QB5, BA1–BA4, BA6–BA9, and BA11–BA14) are small holder breeders, having less than 10 heads in each farm, 3 farms (LQ1, BA5, and BA10) are semi-intensive farms (over10 to 50 heads) and both of these small farms and the semi-intensive ones raring cattle of Native and Mixed breeds, and the other 4 farms (QC6, FT1, BA15, and DZ1) are intensive farms, having a cattle herd of more than 300 heads, rearing Holstein cattle except for farm BA15 that has few number of Simmental cattle rearing together with Holstein cows*.

Genomic DNA was extracted from 300 μl whole blood using the Wizard Genomic DNA Purification Kit (Promega; Madison, WI, USA), following manufacturer's instructions. The concentration of extracted DNA samples was measured using NanoDrop One Spectrophotometer (Thermo Fisher Scientific; Waltham, MA, USA). DNA samples were diluted in nuclease-free water to a final concentration of 30 ng/μl for PCR experiments.

### Evaluation of BLV PVL Using CoCoMo-qPCR-2

BLV PVL was estimated using CoCoMo-qPCR-2 (RIKEN Genesis; Kanagawa, Japan), as described previously (7, 13). Briefly, a 183-bp sequence of the BLV LTR regions was amplified in a reaction mixture containing THUNDERBIRD Probe qPCR Mix (Toyobo; Tokyo, Japan), using the degenerate primer pairs, CoCoMo FRW and CoCoMo REV, and a 15 bp 6-carboxyfluorescein (FAM)-labeled LTR probe. To normalize the viral genomic DNA level within the host cellular genome, a 151-bp sequence of *BoLA-DRA* was amplified using the primer pairs, DRA-FW and DRA-RW, and a FAM-labeled DRA probe, as previously described (13). The PVL was calculated using the equation (number of BLV-LTR copies/number of *BoLA-DRA* copies) × 10^5^ cells.

### Nested PCR Amplification of BLV *env*-gp51 Gene Fragment and Nucleotide Sequencing

A 598-bp fragment of BLV *env*-gp51 gene was amplified via nested PCR using Prime STAR GXL DNA Polymerase (Takara Bio Inc.; Otsu, Japan) as described previously (29–31). The primer set External Forward (5′-ATGCCYAAAGAACGACGG-3′) and External Reverse (5′-CGACGGGACTAGGTCTGACCC-3′) resulted in the first-round amplification of the 913-bp fragment of full length BLV *env* gene corresponding to the nucleotide positions 4826 to 5738 of the whole BLV genomic sequence recorded in GenBank (accession No. EF600696) (32), and then internal ENV_5032_ (5′- TCTGTGCCAAGTCTCCCAGATA-3′) and internal ENV_5608r_ (5′-AACAACAACCTCTGGGAAGGGT-3′) resulted in the second-round amplification of the 598-bp fragment of the BLV *env*-gp51 gene corresponding to the nucleotide positions 5037 to 5634 of the whole BLV genomic sequence.

Positive second-round PCR products were purified using 5 × Exo-SAP IT (USB Corp.; Cleveland, OH, USA) and directly sequenced on an ABI3730x1 DNA Analyzer using ABI PRISM BigDye Terminator v3.1 Ready Reaction Cycle Sequencing Kit (Applied Biosystems; Foster City, CA, USA) using the primers ENV_5032_ and ENV_5608r_. The resulting sequences included a 501-bp region of the *env* gene, corresponding to nucleotide positions 5084 to 5584 of the whole BLV genomic sequence. The editing, alignment, and identification of nucleotide sequences were performed using MEGA 7 software (33).

### Phylogenetic Analysis and Phylogenetic Tree Construction

The partial BLV *env-*gp51 sequences from the Egyptian isolates were aligned with the 51 partial BLV *env-*gp51 sequences from GenBank (representative of the 11 BLV genotypes distributed worldwide) using MEGA 7 software. Phylogenetic analysis of the partial BLV *env*-gp51 sequences from 50 BLV positive samples successfully amplified using nested PCR were also conducted using MEGA 7 software (33). For robust and accurate phylogenetic analysis of the BLV *env-*gp51 sequence, the “find best DNA/Protein models” tool of MEGA 7 software was used to choose the best fit model. The Kimura-2 parameter model was chosen as the model with the best fit to analyze the BLV *env-*gp51 sequence with the smallest Akaike information criterion (AIC) value. Two phylogenetic trees were constructed using the maximum likelihood (ML) algorithm based on 420 and 501 bp sequences with the K2+I and K2 models of nucleotide substitution in MEGA 7, respectively. The reliability of the phylogenetic relationships was evaluated using non-parametric bootstrap analysis with 1,000 replicates. The deduction of protein sequence through translation of nucleotide to amino acid sequence was performed using MEGA 7 (33). The sequences of isolates obtained in this study were deposited in GenBank under accession numbers: LC498589 (DZ1.1), LC498590 (DZ1.2), LC498591 (DZ1.3), LC498592 (QB1.1), LC498593 (QB3.1), LC498594 (QB5.1), LC498595 (QC6.1), LC498596 (QB2.1), LC498597 (QB4.1), LC498580 (DZ1.4), LC498581 (DZ1.5), LC498582 (DZ1.6), LC498583 (QB1.2), LC498584 (QB3.2), LC498585 (QB5.2), LC498586 (DZ1.39), LC498587 (DZ1.40), LC498588 (QB5.3), and LC500799~LC500830 (DZ1.7~DZ1.38).

### Statistical Analysis

The significance of PVL difference between the cattle from different geographic regions was tested using multiple comparison by Tukey's test after the analysis of variance. *P* < 0.05 was considered significant.

## Results

### Prevalence of BLV Among Egyptian Cattle

Blood samples were collected from 270 dairy cattle of 24 farms located at five provinces of different geographic location in Egypt (Figure 1). Cattle samples were classified as Native Egyptian (12 samples), Holstein (125 samples), Mixed (129 samples), and Simmental (4 samples) cattle (Table 1). All samples were tested for BLV infection using BLV-CoCoMo-qPCR-2 assay, which detects the two copies of BLV LTRs present per provirus. Our results showed that BLV infection rate varied among the cattle tested from farms in different regions (Table 1). Interestingly, BLV infection was detected only in the cattle from two provinces (Qena and Damietta), while those from the other three provinces (Beheira, Luxor, and Fayoum) tested BLV-negative. In Qena province, 74 blood samples were collected from the cattle of six farms, namely, QB1, QB2, QB3, QB4, QB5, and QC6. Five (QB1, QB2, QB3, QB4, and QB5) are small farms (<10 cattle) located at El-Brahma town, harboring either Native or Mixed breed cattle, while the sixth (QC6) is a large farm (~300 cattle) located at the provincial capital town, harboring only Holstein breed cattle. It was interesting that the cattle from all 6 farms were BLV-positive and the infection rate for each distinct breed was 0–60% (Table 1). In Damietta province, 40 blood samples were collected from the cattle of a large intensive farm (DZ1) located at El-Zarqa harboring ~500 cattle of Holstein breed. Surprisingly, all the tested samples were BLV-positive, thus having 100% infection rate (Table 1). A total of 137 samples were collected from the cattle of the 15 farms (BA1–BA15) located at Abu-Elmatamir in Beheira province, among which 14 are small or semi-intensive farms harboring Native or Mixed breeds and BA15 is a large intensive farm harboring Holstein and Simmental cattle. All the tested samples were BLV-negative, thus having 0% infection rate for each farm. In Luxor province, the 16 samples collected from Native and Mixed cattle in one small farm (LQ1) located at EL-Qurna were BLV-negative. A limited number of available samples from Holstein cattle in one large dairy farm (FT1) from Fayoum province was tested, but none of them were BLV-positive.

Our results demonstrated that 58 samples among the 270 tested samples were BLV-positive (21.5%; Table 1 and Figure 1) and BLV infection was distributed only in two provinces; thus, BLV infection in Egypt may be limited to particular regions.

### Phylogenetic Analysis of the 420-bp Sequence of BLV *env-*gp51 Region of the Selected 18 Typical BLV Strains in Egypt and Known Strains From Different Geographic Locations Worldwide

To analyze the genetic variability among the BLV strains in Egypt, phylogenetic characterization was carried out after sequencing the BLV *env-*gp51 gene. Out of the 58 BLV-positive samples collected from the cattle in six different farms in Qena province and one farm in Damietta province as shown in Table 2, 50 samples were successfully amplified by nested PCR for partial BLV *env-*gp51 gene and a 501-bp sequence corresponding to the nucleotide positions 5084 to 5584 of the full-length BLV genome of the reference strain FLK-BLV subclone pBLV913 (accession number EF600696) were obtained.

**Table 2:** Distribution of the six genetically distinct BLV strains circulating in Egypt cattle throughout the infected dairy farms.

**Province**	**Town**	**Farm ID**	**BLV infectivity by CoCoMo-qPCR (%)**	**BLV isolated strains**						
				**G-1**				**G-4**		**No. of detected isolates by nested PCR**
				**a**	**b**	**c**	**d**	**x**	**y**	
Qena	El-Brahma	QB1	3/10 (30.0)	–	1^m^	–	–	1 ^m^	–	2
		QB2	1/4 (25.0)	–	–	1^m^	–	–	–	1
		QB3	3/5 (60.0)	–	1 ^m^	–	–	1 ^m^	–	2
		QB4	2/7 (28.6)	–	–	–	1^n^	–	–	1
		QB5	4/9 (44.4)	–	1 ^m^	–	–	1 ^n^	1^m^	3
	Qena-city	QC6	5/39 (12.8)	–	1 ^h^	–	–	–	–	1
Damietta	El-Zarqa	DZ1	40/40 (100.0)	3^h^	–	–	–	35 ^h^	2 ^h^	40
Total				3	4	1	1	38	3	50

m *Mixed breed*;

n *Native breed*;

h *Holstein breed*.

**Figure 2:**
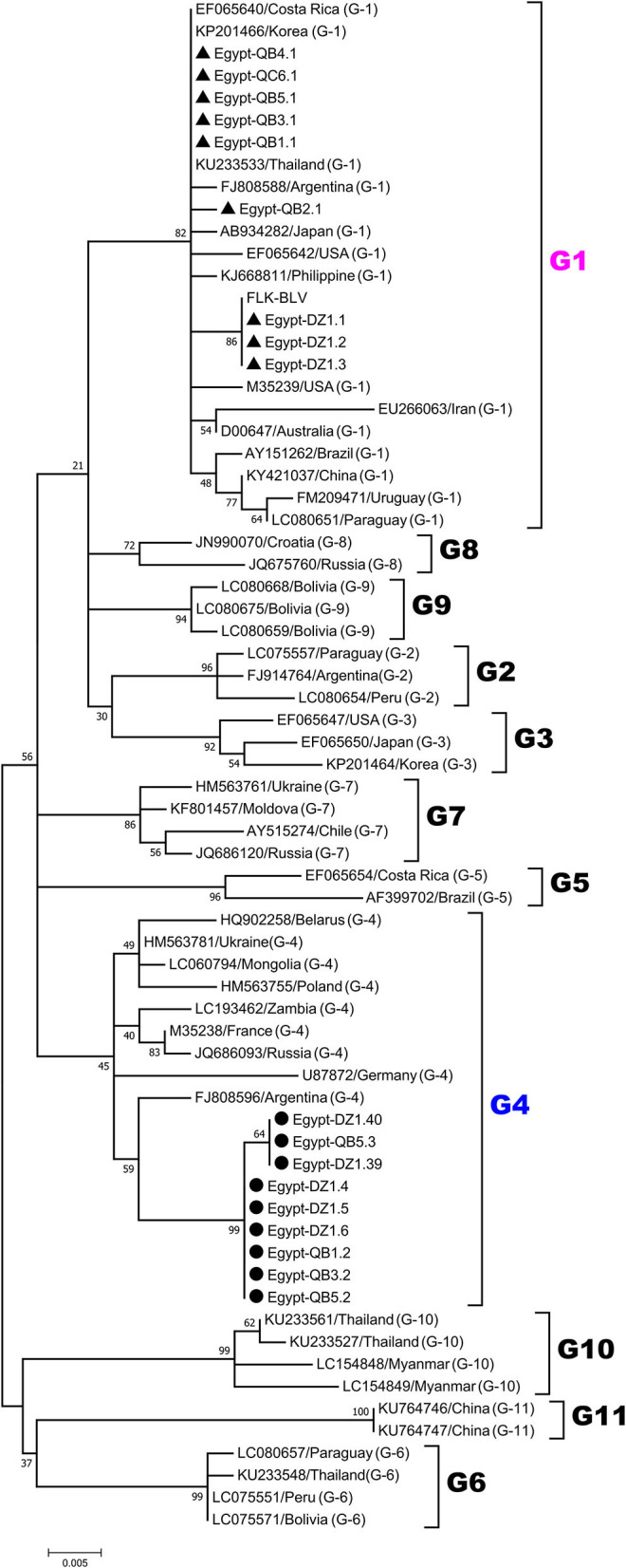
Maximum likelihood (ML) phylogenetic tree based on 420-bp nucleotide of partial BLV *env*-gp51 gene sequences from the selected 18 typical BLV isolates in Egypt and 51 BLV isolates from other countries worldwide. The Egyptian isolates are indicated by the country name together with the sample ID and farm origin. Other isolates are indicated by the accession number and country name. Egyptian BLV isolates are aligned as shown in Figure 4, and those clustered into genotypes-1 and-4 are marked by filled triangles (▴) and circles (•), respectively. BLV genotypes are indicated by numbers on the right side of the tree. The bottom bar of the tree denotes distance.

For phylogenetic analysis of BLV genotypes in Egypt, the 420-bp sequences corresponding to the nucleotide positions 5126 to 5545 of the full-length BLV genome were used in order to be shared with all the currently known 11 genotypes of BLV in the world to our references. The 18 selected BLV isolates out of the 50 PCR-amplified samples were aligned with those from BLV strains representing all known 11 different BLV genotypes (genotype-1 to-11) deposited in GenBank. An ML phylogenetic tree was constructed using the best K2+I model of nucleotide substitution (33). The results of phylogenetic analysis were similar to those published in previous studies and BLV strains were classified into 11 genotypes (3, 18, 29, 34–36), as shown in Figure 2. Interestingly, our phylogenetic analysis also showed that the Egyptian BLV isolates were clustered into genotype-1 or -4.

### Identification of the Six Genetically Distinct Strains Present in Egyptian Cattle by Phylogenetic Analysis Based on the 501-bp Sequence of BLV *env-*gp51 Region

**Figure 3:**
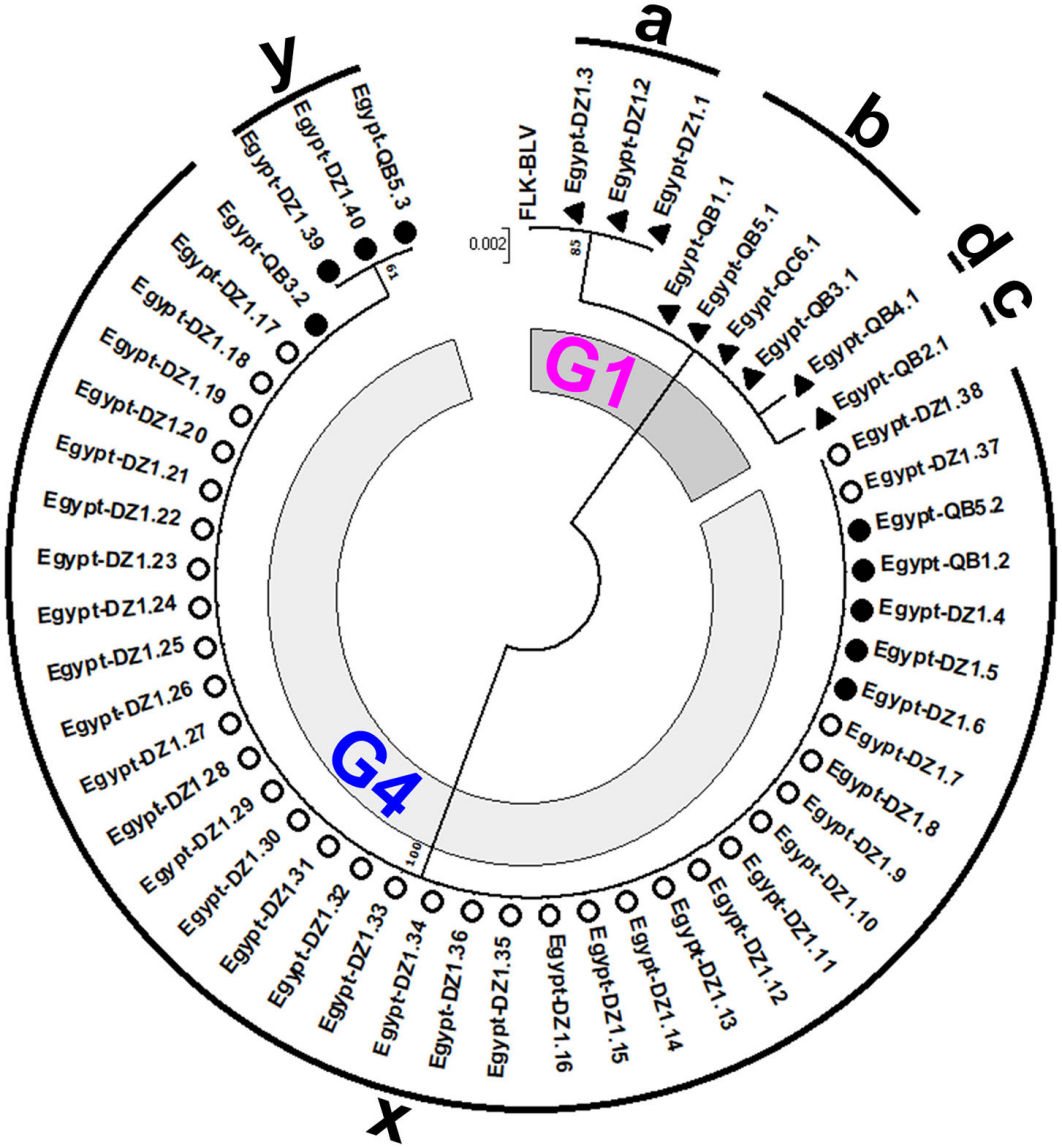
Maximum likelihood (ML) phylogenetic tree based on 501-bp nucleotide of BLV *env*-gp51 gene from 50 BLV isolates classified into the six genetically distinct strains identified in Egyptian cattle (G1-a, G1-b, G1-c, G1-d, G4-x, and G4-y). This tree was constructed with the sequences of 50 BLV isolates detected in the cattle from different farms at two geographic locations in Egypt, including the 18 typical BLV Egyptian isolates as shown in Figure 2 and other Egyptian isolates detected in different locations, and the reference strain FLK-BLV subclone pBLV913 (accession number EF600696). Egyptian BLV sequences on the tree are indicated by farm ID, and sample number. BLV isolates that belong to genotype-1 are indicated using filled triangles (▴), while those that belong to genotype-4 are indicated using open (◦) and filled (•) circles. Sequences for each distinct BLV strain are indicated via alphabets around the circumference of the figure. The 18 typical Egyptian BLV isolates from identical samples were aligned as shown in Figure 4 and are indicated by filled circles (•) and triangles (▴). Other isolates are indicated using open circles (◦). The genotypes are shown as G1 and G4. The bar at the above portion denotes distance.

To gain insight into the genetic variability of BLV strains present in Egyptian cattle, we classified all the 50 BLV isolates via another ML phylogenetic tree based on the 501-bp sequence of BLV *env*-gp51 region (Figure 3). In this tree, all the 50 BLV isolates were aligned with the reference strain. It clearly showed that nine out of 50 BLV sequences belonged to genotype-1 and were further classified into four distinct strains (G1-a, G1-b, G1-c, and G1-d). In contrast, the remaining 41 BLV isolates belonged to genotype-4 and were further classified into two distinct strains (G4-x and G4-y). Thus, our results indicate that six genetically distinct BLV strains are present in Egyptian cattle.

### Nucleotide and Amino Acid Sequence Analysis of the 501-bp Sequence of BLV *env*-gp51 Region of the Six Genetically Distinct Strains Present in Egyptian Cattle

The similarity of the 501-bp sequence of BLV *env*-gp51 region of all 50 Egyptian isolates ranged from 96.7 to 100% (data not shown). The sequence from the nine Egyptian BLV isolates belonging to G1-a, G1-b, G1-c, and G1-d strains exhibited the highest similarity (96.7–100%) to that from all BLV strains representing genotype-1 from various geographic locations worldwide used as references for the phylogenetic analysis (data not shown). In contrast, the sequence from the 41 Egyptian BLV isolates belonging to G4-x and G4-y strains were 97.7–99.1% similar to that from all BLV strains representing genotype-4 (data not shown).

**Figure 4:**
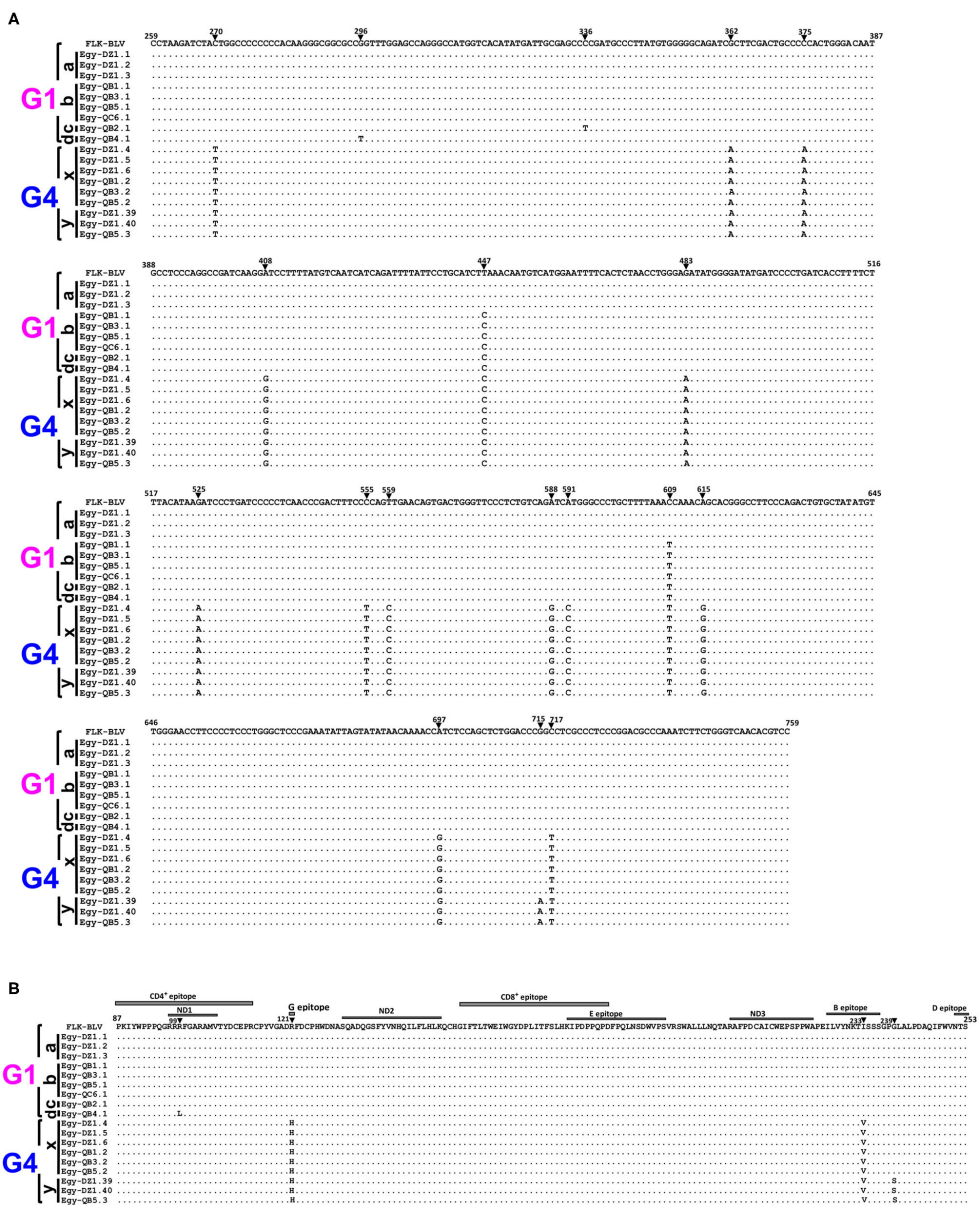
Alignment of nucleotide sequences and deduced amino acid sequences of 501-bp nucleotide of BLV *env*-gp51 gene from the six genetically distinct strains identified in Egyptian cattle. Alignment was performed for the selected 18 typical Egyptian BLV isolates representative for the six genetically distinct strains circulating in Egyptian cattle (G1-a, G1-b, G1-c, G1-d, G4-x, and G4-y). Egyptian BLV isolates are shown by the country name abbreviation (Egy), farm ID, and sample ID. **(A)** Nucleotide sequence alignment showing the nucleotide substitutions are indicated using numbers and filled triangles above the sequences. **(B)** The deduced amino acid alignment showing the amino acid substitutions are indicated using numbers above the sequences; the labeled gray rectangles refer to coding sequences of the antigenic determinants of the gp51 protein. The black bars at the left side of the figure indicate Egyptian BLV isolates that belong to each particular strain. Genotypes (G1 and G4) are indicated using black braces at the far-left side of the figure. Dots indicate identity with FLK-BLV subclone pBLV913 (accession No. EF600696), which was used as a reference in a reference in this work.

Nucleotide sequences for the 18 typical selected BLV isolates representing the six genetically distinct strains (G1-a, G1-b, G1-c, G1-d, G4-x, and G4-y) were aligned with that of the reference strain (Figure 4). The three sequences for G1-a strain showed 100% similarity with that of the reference strain. The four sequences for G1-b strain exhibited two silent nucleotide substitutions: one located in the third base of residue 149 (nt 447) and the other in the third base of residue 203 (nt 609). We found only one sequence for G1-c strain, which exhibited three nucleotide substitutions: two of them were similar to those in G1-b strain sequences, while the third was located in the third base of residue 112 (nt 336); however, all of them were silent substitutions. Similarly, we detected one sequence for G1-d strain, which exhibited three nucleotide substitutions: two of them were silent and at the same positions as those in G1-b strain sequences, while the third one was a G to T substitution in the second base of residue 99 (nt 296), which changed the deduced amino acid located in the neutralizing domain (ND1) and the CD4^+^ epitope region of gp51 protein from glycine to lysine. We found 15 nucleotide substitutions for G4-x strain. Among them, 13 were silent and included residues 90 (nt 270), 125 (nt 375), 136 (nt 408), 149 (nt 447), 161(nt 483), 175 (nt 525), 185 (nt 555), 187 (nt 559), 196 (nt 588), 197 (nt 591), 203 (nt 609), 205 (nt 615), and 239 (nt 717), but the other two substitutions were not silent. The first substitution (G to A) occurred in the second base in residue 121 (nt 362), changing the deduced amino acid located in the epitope G of gp51 protein from arginine to histidine, while the second substitution (A to G) in the first base in residue 233 (nt 697) changed the amino acid located in the B epitope region from isoleucine to valine. The substitutions for G4-y strain were identical to those for G4-x strain; however, one additional non-silent substitution (G to A) in the first base of residue 239 (nt 715) was detected, which changed the deduced amino acid from glycine to serine, which is a non-synonymous residue of gp51 protein.

### Distribution of the Six Genetically Distinct Strains Throughout the Infected Farms in Egypt

We summarized the distribution of the six genetically distinct strains at different farms to specify the strain present in the cattle from each BLV-infected farm (Table 2). Strain G1-a was detected in three different cattle from DZ1 farm located at El-Zarqa in Damietta province. Strain G1-b was found in four different cattle: three isolates were detected as identical in the cattle from QB1, QB3, and QB5 farms located very close to each other at El-Brahma in Qena province, while the fourth isolate was detected in the cattle from QC6 farm located in the provincial capital town. One isolate of strains G1-c and G1-d each were detected in the cattle from QB2 and those from QB4 farm, respectively, both located at El-Brahma. Among the 38 isolates classified as G4-x strain, 35 were detected in the cattle from DZ1 farm located at El-Zarqa in Damietta province and one each were detected in cattle from QB1, QB3, and QB5 farms. Among the three isolates classified as G4-y strain, two were detected in the cattle from DZ1 farm and the third was detected in those from QB5 farm.

Regarding the distribution of the six genetically distinct strains present in Egyptian cattle, our results concluded that genotype-1 and-4 strains coexisted in most BLV-infected farms.

### Estimation of BLV PVL Among Egyptian Cattle

**Figure 5:**
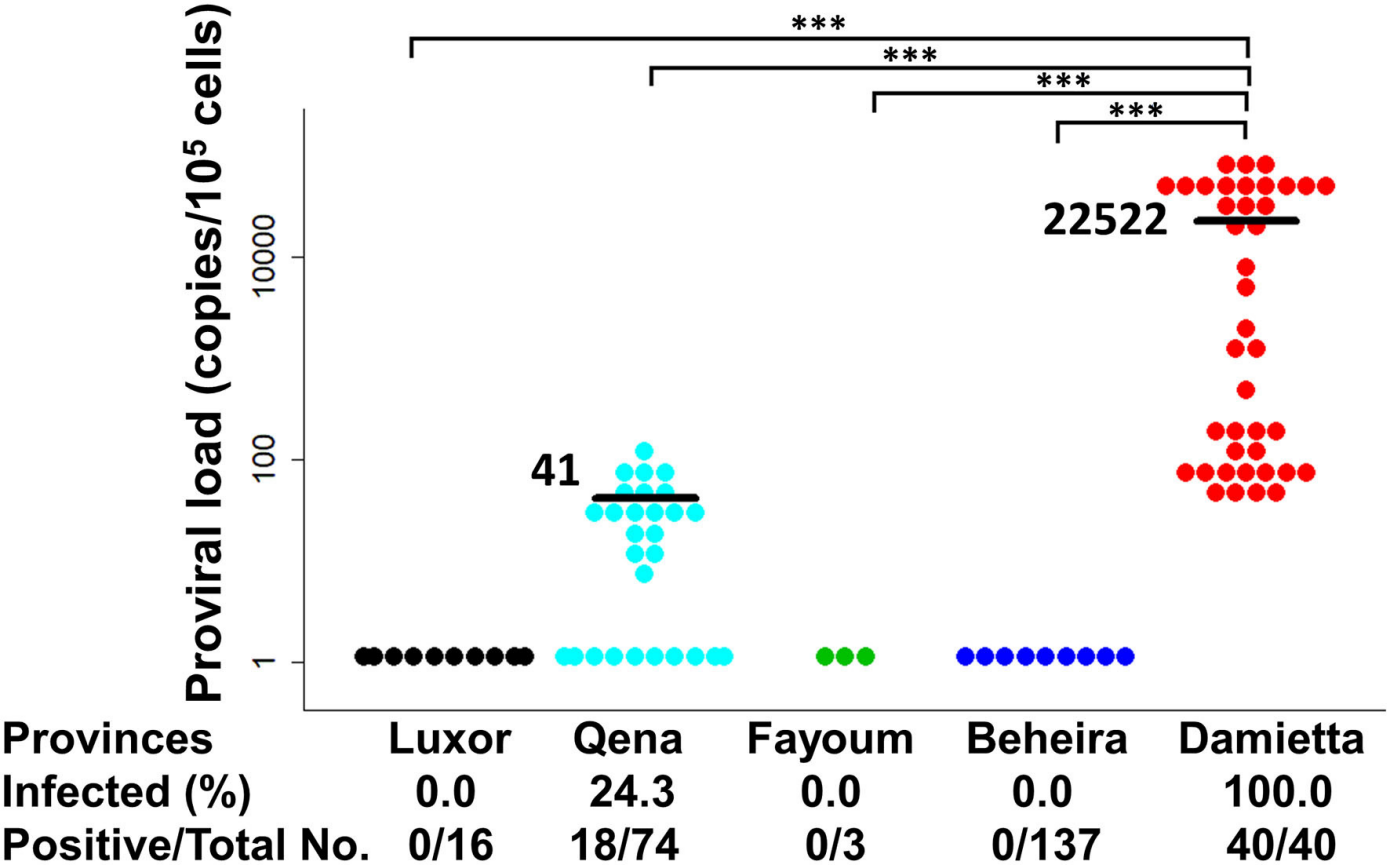
Estimation of BLV proviral load (PVL) among Egyptian cattle. BLV PVL calculated using CoCoMo-qPCR-2 among cattle samples were tested in five different provinces in Egypt. Black bars show the main BLV PVL values. The actual number (No.) of cattle reported positive via BLV-CoCoMo-qPCR-2 per total No. of tested cattle and BLV-infected rate (%) are indicted at the bottom of each block. Significant differences between different geographic regions were calculated using Tukey's test. *P*<0.05 was considered significant. ***P < 0.00.

PVL is an important risk factor of BLV-associated disease progression and transmission risk (7–12). Therefore, BLV PVL was calculated by CoCoMo-qPCR2 assay and its level was summarized with BLV infection rate in each tested province of Egypt, as shown in Figure 5. In Qena province, 18 out of the 74 tested samples were BLV-positive, their PVL ranging from 7 to 125 copies/10^5^ cells with a mean value of 41 copies/10^5^ cells. In DZ1 farm from Damietta province, all 40 tested samples were BLV-positive and the PVL ranged from 45 to 98,725 copies/10^5^ cells with a mean value of 22,522 copies/10^5^ cells. Our previous reports (11, 12) suggest that a PVL of around 10,000 copies/10^5^ cells in cow blood might be an indicator of efficient BLV spreading within the whole body, thereby easily detecting BLV proviruses into milk, nasal mucus, and saliva. Interestingly, 17 cattle from DZ1 farm (42.5%) had high PVL (>10^4^ copies/10^5^ cells) ranging from 16,985 to 98,725 copies/10^5^ cells, indicating that they may be high-risk transmitters. Indeed, all tested samples from DZ1 farm were BLV-positive with infection rate of 100% (Table 2). In addition, 15 of these 17 cattle were infected with G4-x strain and 2 with G4-y strain, as shown in Table 2. In contrast, all 18 BLV-positive cattle from 5 different farms in Qena province had low PVL (≤10^4^ copies/10^5^ cells).

## Discussion

This study provides a new molecular evidence for BLV infection in Egyptian cattle. First, we successfully detected BLV provirus, which integrates into the host genome, in the blood of BLV-infected cattle with a prevalence rate of 21.5%, since the previous studies focused only on the serological detection of BLV in Egyptian cattle. BLV infection was detected in cattle from dairy farms in two of the five tested provinces, suggesting that BLV infection might exist in only particular regions. However, it is possible that the absence of BLV infection in the other three regions resulted from the small number of samples investigated. Therefore, further studies are required to accurately determine the prevalence of infection through a nation-wide survey. Second, the phylogenetical analysis and alignment of the partial BLV *env*-gp51 sequences clearly showed at least six genetically distinct strains, which belong to genotype-1 and-4 among the 11 globally detected genotypes, are present in Egyptian cattle. This is the new report of BLV genetic diversity in Egypt through molecular characterization. Third, we concluded that BLV PVL level is associated with viral horizontal transmission under field conditions in Egypt. Interestingly, in DZ1 farm, where all collected samples were BLV-positive, 35 among the 40 cattle were infected with G4-x strain and the PVL in 15 among these 35 BLV-infected cattle was >10,000 copies/10^5^ cells, indicating they may be high-risk transmitters. We demonstrated that BLV-infected cattle with high PVL are a source of infection in BLV-free cattle in Egyptian dairies, which supports the results of previous studies (7–12, 37). In addition, this result supported the previous report that major strain was the source spread at the farm, which existed several BLV strains (38).

Egypt is located between the three continents of Africa, Asia, and Europe. Most of the neighboring countries of Egypt are BLV-infected, including Greece (3), Turkey (39), Jordan (40), Syria (41), and Saudi Arabia (42), however there is no information for the disease in several neighboring countries including Libya and Sudan (43). The total percentage of BLV prevalence determined in our study was 21.5%. This result is in agreement with the results of the recent seroprevalence study reporting BLV infection rate to be 20.8% among Egyptian cattle (28) and similar to the previously reported BLV infection prevalence rate of 15.83% (27). Likewise, BLV infection rate reported in some of the neighboring countries include 20.2% in Saudi Arabia (42) 7.75% in Iraq (44), despite being high (48.3% in herd level) in Turkey (39). In contrast, the BLV infection rate among cattle of Egypt reported in this study was close to or lower than that in India (27.9%) (45). However, the BLV infection rate reported here compared with that reported in some other Asian countries was being higher than that reported in Philippines (4.8–9.7%) (29) and markedly lower that in Japan (40.9%) (46), Korea (42.16%) (47), and Myanmar (37.04) (36). The Egyptian livestock import has increased over the last 10 years and the Egyptian dairy industry is completely dependent on imported cattle for herd construction. Germany, Netherlands, and the United States of America represent the major cattle suppliers in Egypt (23). Indeed, phylogenetic analysis of the total 50 partial *env*-gp51 sequences detected here clearly showed that Egyptian BLV isolates were clustered into genotype-1 and-4, which have been detected worldwide. Genotype-1 is the most dominant genotype of BLV, distributed across almost all continents, including Europe, America, Asia, and Australia. Genotype-4 is the second most widely distributed genotype, primarily detected in Europe and some American countries (3). Noticeably, genotype-1 and-4 cover large geographic areas from Europe to America, suggesting the possibility of extensive trading between countries (47). Therefore, it is recommended to determine the risk factors for BLV infection that might have been introduced to Egypt from the imported cattle breeds or be specific for the management system employed for cattle in the field.

The phylogenetic analysis of the 420-bp sequences of *env-*gp51 region in this study showed that Egyptian BLV isolates were assigned to genotype-1 and-4 and alignment of the 501-bp sequences of *env-*gp51 region resulted in the identification of 18 nucleotide substitutions, of which 14 were silent and four were amino acid substitutions. The substitutions varied according to genotype. For example, phylogenetic tree based on the 501-bp sequence of *env-*gp51 region demonstrated four different strains (G1-a, G1-b, G1-c, and G1-d) with genotype-1 and two different strains (G4-x and G4-y) with genotype-4. Similarly, Marawan et al. (37) performed phylogenetic analysis and demonstrated the existence of 20 different BLV subgenotypes in Miyazaki prefecture of Japan based on their nucleotide sequences. Interestingly, the substitutions in genotype-1 strains were highly conserved and only one unique non-synonymous substitution was analyzed in the G1-d strain sequence, which is located at the residue 99 and changed an amino acid in the neutralizing domain (ND1) and the CD4+ epitope region of gp51 protein from glycine to lysine. Moreover, in the genotype-4 strains, G4-x strain exhibited two amino acid substitutions in residues 121 and 233, which were located in G and B epitopes, respectively, and G4-y strain exhibited three substitutions, two of them were identical to the aforementioned substitutions in G4-x strain and the third was an unique amino acid substitution in residue 239, which is located in the region between B and D epitopes of gp51 protein.

In this study, we noticed that, in a single farm DZ1, all the 40 samples collected were BLV-positive, with 35 of these BLV isolates being identical and classified as the same BLV strain (G4-x) with genotype-4. Two isolates were classified as G4-y strain having a 99.8% similarity with G4-x strain, but the remaining three isolates were classified as G1-a strain with genotype-1. Interestingly, as calculated by CoCoMo-qPCR2, BLV PVL of these BLV-infected cattle ranged from 45 to 98,725 copies/10^5^ cells with a mean value of 22,522 copies/10^5^ cells. Regarding horizontal BLV transmission, we previously detected BLV provirus in the nasal secretions and saliva of cattle with PVL >14,000 and >18,000 copies/10^5^ cells in blood, respectively, and suspected that BLV-infected cells were present in nasal secretions and saliva, respectively (11). Furthermore, the BLV provirus was detected in milk samples from dams when the PVL in blood samples were approximately >10,000 copies/10^5^ cells (12). It might infect healthy cattle via licking, sneezing, rubbing of noses, or milking. In this study, the PVL in 15 among the 35 infected cattle harboring G4-x strain in farm DZ1 appeared to be >10,000 copies/10^5^ cells, indicating that they may be high-risk transmitters. Therefore, we hypothesized that the infected cattle with high PVL act as virus spreader and transmit genetically identical provirus to other cattle during long-time physical contact. Thus, the G4-x strain is widely spread in cattle from DZ1 farm. This result harmonized with those of Murakami et al. (38), who showed that three genetically distinct BLV strains co-existed among the infected cattle in one farm and described that the major viral strains were the source of infection spread in that farm. In contrast, BLV-infected cattle with low PVL are not a source of infection for BLV-free cattle (14). Therefore, it is recommended to isolate and monitor the BLV-infected cattle with high PVL to prohibit virus spread horizontally and vertically.

Here we detected BLV infectivity in two provinces: Qena and Damietta. We examined cattle from six dairy farms in Qena province and found cattle from all of them to be BLV-infected. Five of these farms (QB1, QB2, QB3, QB4, and QB5) are small-sized, but farm QC6 is large. The infection rate was higher in the small-sized farms than that in the large farm (Table 2). These findings concurred with those of other studies demonstrating that average BLV seroprevalence in small farms (less than 20 cattle) was higher than that in medium or large farms in Miyazaki prefecture, Japan (48). Several studies have described the risk factors for BLV transmission within herd and considered that physical contact (49), loose housing system (50), use of common sleeves during rectal palpation (51), blood sucking insects, blood-contaminated devices, and needle reuse (4) are the most dominant factors for virus transmission. Therefore, it is possible that in the small-sized farms, loose cattle housing system, presence of hematophagous insects, and poor managemental procedures may increase the chance for virus transmission from infected to uninfected cattle.

In contrast, in DZ1 farm located in Damietta province, we demonstrated the highest BLV infection rate (100%). It is a large dairy farm (~500 cattle) and depends on natural breeding for reproduction. Interestingly, the milking system of this farm depends on using a limited number of common portable milking machines, thus lacking sufficient cleaning during milking between individual cows. Therefore, the likelihood of bovine milk pathogen transmission is higher in herds using mobile milking machines than that in herds using fixed milking machines, as previously demonstrated (52). In addition, BLV has a broad tropism to the mammary tissue (53) and the milk or milk cells of infected cattle could be a source of infection (12, 54, 55). However, the role of milk in virus transmission is considered to be little (56). Thus, we hypothesized that there is a possibility for BLV transfer from infected to healthy receptive cattle during the milking process.

To gain insight into the fundamental nature of existence of each genetically distinct virus strain, we checked the virus strain spreading the infection between animals in each particular farm. In the five small-sized BLV-infected farms (QB1, QB2, QB3, QB4, and QB5) harboring Native and Mixed breed cattle located in El-Brahma, Qena, the BLV infection rate was ranged from 25 to 60%. These farms were neighbors and located very close to each other. Shettigara et al. (57) have proposed that at least 200 m distance should be maintained between farms to prevent transmission among herds. It is interesting that the cattle from three of these farms (QB1, QB3, and QB5) harbored genetically identical strains (G1-b and G4-x), as shown in Table 2. This result might be a good example for horizontal BLV transmission from one farm to its adjoining farm in the field. On the contrary, we identified two genetically different strains G1-c and G1-d in farms QB2 and QB4, respectively. The sixth farm (QC6) located in the capital town of Qena province was a large-sized dairy farm (~300 cattle) harboring Holstein breed cattle. Although the BLV infection rate in this farm was 12.8% (5/39) as determined by CoCoMo-qPCR, we successfully identified a single BLV isolate classified as G1-b strain. This might be due to the difference in sensitivity between CoCoMo-qPCR assay used for virus detection and nested PCR technique used for gp51 amplification (29).

In conclusion, the phylogenetic analysis based on partial BLV *env-*gp51 sequence revealed that there are at least six genetically distinct BLV strains present in Egyptian cattle, which belong to genotype-1 and-4 among the 11 globally detected genotypes. This study provides a new molecular characterization of BLV in Egyptian dairy cattle and calculated the PVL among BLV-infected cattle in Egypt. This study also showed that the BLV infection prevalence was 21.5% (58 out of 270 head) in five provinces located in the northern, central, and southern parts of Egypt. Further research should focus on determining the impact of BLV genetic diversity on viral pathogenicity and disease progression in dairy cattle on a large scale.

## Data Availability Statement

The datasets presented in this study can be found in online repositories. The names of the repository/repositories and accession number(s) can be found in the article/supplementary material.

## Ethics Statement

All animals were handled by the regulation of the Animal Ethics Committee at the Faculty of Veterinary Medicine, South Valley University, Qena, Egypt, and by the regulation of RIKEN, Japan in strict accordance with good animal practice following the guidelines of RIKEN. The study was reviewed and approved by Research Code of Ethics (RCOE-SVU) at the South Valley University, and by the RIKEN Animal Experiments Committee (approval number H29-2-104).

## Author Contributions

YA conceived of and designed the study. SM, AA, AA-H, and AM collected the samples. RH, SM, MP, LB, SW, and YA acquired, analyzed, and interpreted the data. YA contributed reagents, materials, and analysis tools. RH and YA drafted and revised the manuscript. All authors agree to be accountable for the content of the work.

## Conflict of Interest

The authors declare that the research was conducted in the absence of any commercial or financial relationships that could be construed as a potential conflict of interest.
